# Supplementary material for: The Contest of Nanoparticles: Searching for the Most Effective Topical Delivery of Corticosteroids
Source: Pharmaceutics. 2023 Feb 3;15(2):513. doi: 10.3390/pharmaceutics15020513 (PMC9962773; doi:10.3390/pharmaceutics15020513)
Supplement: Supplementary file 1 [file pharmaceutics-15-00513-s001.zip › pharmaceutics-2101792-supplementary.pdf]

pharmaceutics MDPI

Supplementary Materials: The contest of nanoparticles: Searching for the most effective topical delivery of corticosteroids

Aneta Kalvodová [1], Kristýna Dvořáková [1], Eliška Petrová [1], Bozena B. Michniak-Kohn [2] and Jarmila Zbytovská [1,3,*]

[1] Department of Organic Technology, Faculty of Chemical Technology, University of Chemistry and Technology Prague, Technická 5, 166 28 Prague, Czech Republic
[2] Center for Dermal Research (CDR), Life Sciences Building, Rutgers University, Piscataway, NJ 08854, USA
[3] Department of Pharmaceutical Technology, Faculty of Pharmacy, Charles University, Heyrovského 1203, 500 05 Hradec Králové, Czech Republic
* Correspondence: zbytovsj@vscht.cz

S1. Freeze-drying process for polymeric nanoparticles

The method of lyophilization or freeze-drying is commonly employed for the prolongation of nanoparticle stability upon storage. We used the method to conserve the polymeric nanoparticles and provide the material that is available for re-dispersion prior any experiment after long-term storing. The lyophilization was carried out using AdVantage 2.0 benchtop freeze dryer (SP Scientific, Stone Ridge, US). The method (Table S1) provided uniformly dried lyophilizate with residual moisture < 3 %. The moisture was determined gravimetrically by subtracting the theoretical weight of the nanocarriers from the weight of obtained lyophilizate using the following equation:

$$\text{Residual moisture (\%)} = \frac{w_{(\text{lyophilizate})} - w_{(\text{nanocarriers})}}{w_{(\text{nanocarriers})}} \times 100$$

The lyophilizate was also tested for the ability to be easily and quickly re-dispersed into system containing nanoparticles with size and polydispersity index similar to the original formulation. It was found that PNP lyophilizate provides suitable formulation after reconstitution by distilled water for 10 minutes of intensive mixing at ambient temperature. The comparison of colloidal characteristics of original and reconstituted formulation is shown in Table 1. From the results it is clear that this way of stability prolongation is suitable for this type of system and the nanoparticles do not lose their initial properties.

Table S1. Freeze-drying method for lyophilization of 10 ml of nanoformulation processed in 1 cm thick layer and comparison of colloidal characteristics of polymeric nanoparticles containing HC or HCB.

Step	Temperature [°C]	Time [min]		Vacuum [mtorr]
Freezing	-40	60	Hold	$760\cdot10^3$
Primary drying	-35	500	Hold	200
Secondary drying	+35	240	Ramp	200
	+35	240	Hold	200
Post heat treatment	+5			500

	Initial characteristics			Reconstituted characteristics			
	Size [nm]	PDI	ZP [mV]	Size [nm]	PDI	ZP [mV]	Residual moisture [%]
HC-PNP	186 ± 4	0.04 ± 0.02	-15 ± 1	194 ± 4	0.08 ± 0.01	-16 ± 3	2.75 ± 0.82
HCB-PNP	182 ± 5	0.03 ± 0.01	-16 ± 2	192 ± 3	0.03 ± 0.02	-6 ± 4	2.67 ± 0.41

S2. Solubility of the drugs in formulation excipients

S2.1 Methods

The solubility of HC and HCB in formulation excipients was determined as follows: a super-saturated solution of the drug was shaken for 48 hours on thermostated laboratory shaker at 32 °C. The undissolved drug was separated from the solution by centrifugation (OHaus, Switzerland) at 12000 rpm for 5 min and the supernatant was appropriately diluted in the mobile phase and analyzed using high performance liquid chromatography (HPLC, section 2.8). The solubility was determined in distilled water, phosphate buffer (PBS, pH 7.4), isopropyl myristate (IPM), LNC lipid phase (mixture of IPM and surfactants in the exact ratio as in LNC), in solution of ethanol and PBS (1:1, v/v) and in 0.5% (for HC) and 2% (for HCB) solution of PVA in distilled water (w/v).

S2.2 Results and discussion

The solubility of hydrocortisone and hydrocortisone-17-butyrate was assessed in solvents and mixtures used in the preparation of the described nanoformulations (Table S2). The measured solubility of both corticoids in distilled water and PBS correlated to literature data [1,2]. As this type of data is usually not very common in formulation research, we took this opportunity to establish the solubility also in IPM, ethanolic-PBS solution (1:1 v/v) and PVA solutions in DW (0.5% for HC and 2% for HCB). The values obtained were in accordance with the nature of the studied drugs. The solubility of HC was overall higher in more hydrophilic media compared to more lipophilic HCB. HC was scarcely soluble in water and PBS (pH 7.4) with only a small difference between the two. IPM as a compound with balanced lipophilicity also did not provide a high solubilizing effect for HC. However, the addition of surfactants used for the LNC preparation (Kolliphor HS15 and Phospholipon 90G) enabled incorporation of higher drug amount, as they help with its dissolution. Ethanolic solutions are commonly used for an enhanced incorporation of an API into pharmaceutical formulations [3] and as expected, the relatively high ethanol content (50%) in the studied formulation ensured a quite high HC dissolution rate. Polyvinyl alcohol is another example of compound or surfactant that can be used as solubilizing agent for drug formulation. Here, we observed the rise in dissolution of HC in 0.5% PVA water solution (v/v) compared to water alone. For HCB the trends were very similar as described above. Due to its more lipophilic nature, its solubility in water and PBS was much lower than for HC and it was practically insoluble in a highly aqueous environment.

Table S2. Solubility values for hydrocortisone (HC) and hydrocortisone-17-butyrate (HCB) in: distilled water (H_2O), phosphate buffer (PBS, pH=7.4), isopropyl myristate (IPM), lipid phase of lipid nanocapsules (LP), 50% ethanol in PBS (v/v, E50), polyvinyl alcohol solution in distilled water: 0.5% for HC, 2% for HCB (PVA). n ≥ 6

	HC						HCB					
	H_2O	PBS	IPM	LP	E50	PVA	H_2O	PBS	IPM	LP	E50	PVA
Mean [mg/ml]	0.36	0.35	0.87	3.63	13.83	0.48	0.07	0.06	1.01	3.61	8.25	0.08
SEM	0.014	0.012	0.004	0.043	0.130	0.012	0.007	0.004	0.049	0.018	0.082	0.002

S3. *In vitro* release study

S3.1 Methods

In order to estimate the release behavior of corticosteroids from various nanoformulations, a release study was conducted. Samples of all three nanoparticulate systems containing the APIs and a 1% suspension of each corticoid in PBS (w/v) was placed in the upper chamber of Mini Slide-A-Lyzer dialysis device (10 kDa MWCO membrane, 15 ml falcon tube type). The receptor medium was PBS of pH 7.4. The tubes were shaken on a shaker at 100 rpm at 32 °C for 24 hours. The receptor medium was sampled in predetermined intervals and the amount of released drug was determined by HPLC.

S3.2 Results and discussion

An important aspect of the (trans)dermal penetration is the ability of the nanocarriers to liberate the incorporated drug [4]. For this purpose, we subjected the prepared formulations to the *in vitro* release study and observed the drug release behaviour of HC and HCB from LNC, PNP, ETZ and 1% PBS suspension (Figure S1). For HC, the liberation profile in the first 10 hours was comparable for all three nanosystems. Only ETZ showed slightly faster release; however only negligibly. In the following hours the difference was more obvious. LNC and PNP resulted in equivalently released percentage of HC. For LNC, it is expected as HC favours the lipophilic environment of the nanocapsule and the barrier for transition into aqueous phase could be limited. Correspondingly, PNP entrap HC by a combination of hydrophilic and hydrophobic interactions and moreover, HC solubility is increased by the presence of PVA. At 24 hours, the highest portion of diffused HC was observed from ETZ. It could be result of different EE of each system. ETZs showed the lowest EE for HC from all of the formulation, but PL as a surfactant favourably influenced the HC solubility; therefore, the drug was readily available for the skin uptake. Compared to the rest, the simple PBS formulation was the least effective in HC release. In the suspension form, HC crystals has to dissolve at first and only then HC can pass through the membrane. This step is overcome in the nanoformulation as the drug dissolution is elevated by various described ways.

The release of HCB through the membrane was quite different from HC. The overall lower released percentage was caused by significantly lower solubility of HCB in PBS. Also, the lipophilic nature of the drug affected the rate it left the carrier. It was well observable in the case of ETZ. In previous case of HC, ETZs released almost all of the drug. Here, HCB was attached to the PL bilayers strongly (also confirmed by higher EE) and did not permeate into the acceptor as intensively. On the other hand, PNP resulted in the highest percentage HCB liberated; however, it must be kept in mind that HCB-PNP contained only half the drug than other formulations. Thus, its effectivity is high when recalculated to the introduced drug amount, but the total released drug was comparable to LNC and ETZ. LNC release was situated between the other two. Again, the low percentage is the result of both low HCB solubility in PBS and high EE in LNCs.

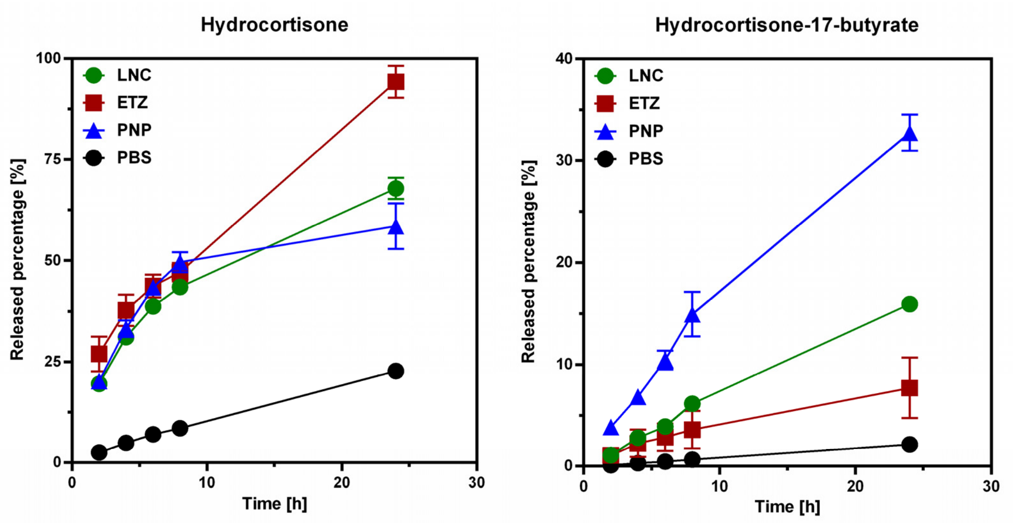

Figure S1. Release behavior of HC and HCB liberated from various nanoformulations (Lipid nanocapsules, LNC; ethosomes, ETZ; and polymeric nanoparticles, PNP) and from a control suspension (PBS) observed for 24 hours. n ≥ 3

S4. FTIR of the skin after treatment by various nanoformulations

FTIR was used as one of the methods for the evaluation of the effect our nanosystems have on the skin barrier. The methodology of this experiment is described in the main body of the article in section 2.5.2. Figure S2 shows the ATR-FTIR spectrum of the human skin with assigned significant peaks. Our area of interest was the position and peak shape of CH_2 symmetrical and asymmetrical vibrations at 2850 and 2915 cm^{-1}, respectively. The positions of said bands were evaluated in the intact and nanoparticle-treated skin and are summarized in Table S3.

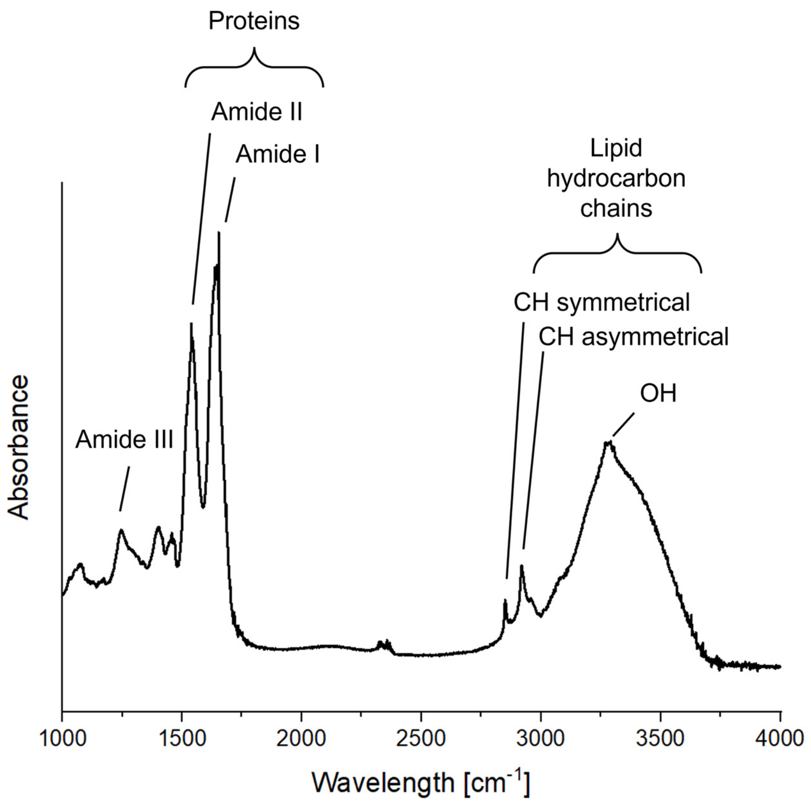

Figure S2. ATR-FTIR spectrum of the human skin with assigned significant peaks.

Table S3. Positions of CH_2 symmetrical ($\upsilon_s CH_2$) and asymmetrical ($\upsilon_{as} CH_2$) vibration peaks in human and porcine skin treated by various formulations.

	Porcine skin		Human skin	
	$\upsilon_s CH_2$	$\upsilon_{as} CH_2$	$\upsilon_s CH_2$	$\upsilon_{as} CH_2$
Intact	2850.1	2918.3	2850.1	2919.4
LNC-treated	2850.2	2918.6	2851.3	2919.9
PNP-treated	2850.1	2918.8	2851.6	2919.9
ETZ-treated	2851.8	2921.2	2852.7	2925.6

References

1. Cavalli, R.; Peira, E.; Caputo, O.; Gasco, M.R. Solid lipid nanoparticles as carriers of hydrocortisone and progesterone complexes with beta-cyclodextrins. *Int J Pharm* **1999**, *182*, 59-69, doi:10.1016/s0378-5173(99)00066-6.
2. Hagen, T.A.; Flynn, G.L. Solubility of Hydrocortisone in Organic and Aqueous Media: Evidence for Regular Solution Behavior in Apolar Solvents. *J Pharm Sci* **1983**, *72*, 409-414, doi:10.1002/jps.2600720422.
3. Ali, H.S.M.; York, P.; Blagden, N.; Soltanpour, S.; Acree, W.E., Jr.; Jouyban, A. Solubility of Budesonide, Hydrocortisone, and Prednisolone in Ethanol + Water Mixtures at 298.2 K. *J Chem Eng Data* **2010**, *55*, 578-582, doi:10.1021/je900376r.
4. Hua, S. Comparison of in vitro dialysis release methods of loperamide-encapsulated liposomal gel for topical drug delivery. *Int J Nanomedicine* **2014**, *9*, 735-744, doi:10.2147/ijn.S55805.
